# Surgical stress and metabolic response after totally laparoscopic right colectomy

**DOI:** 10.1038/s41598-021-89183-7

**Published:** 2021-05-06

**Authors:** Marco Milone, Antonella Desiderio, Nunzio Velotti, Michele Manigrasso, Sara Vertaldi, Umberto Bracale, Michele D’Ambra, Giuseppe Servillo, Giuseppe De Simone, Fatima Domenica Elisa De Palma, Giuseppe Perruolo, Gregory Alexander Raciti, Claudia Miele, Francesco Beguinot, Giovanni Domenico De Palma

**Affiliations:** 1 Department Clinical Medicine and Surgery, Federico II University of Naples, Via Pansini 5, 80131 Naples, Italy; 2 URT Genomics of Diabetes, Institute of Experimental Endocrinology and Oncology, National Research Council, Naples, Italy; 3 Department of Translational Medical Sciences, “Federico II” University of Naples, Naples, Italy; 4 Department of Advanced Biomedical Sciences, “Federico II” University of Naples, Naples, Italy; 5 CEINGE-Biotecnologie Avanzate. INSERM U1138, Centre de Recherche des Cordeliers, Sorbonne Université, Université de Paris, Paris, France; 6 Team “Metabolism, Cancer & Immunity”, Equipe 11, Paris, France

**Keywords:** Biomarkers, Medical research, Molecular medicine, Oncology

## Abstract

No clear consensus on the need to perform an intracorporeal anastomosis (IA) after laparoscopic right colectomy is currently available. One of the potential benefits of intracorporeal anastomosis may be a reduction in surgical stress. Herein, we evaluated the surgical stress response and the metabolic response in patients who underwent right colonic resection for colon cancer. Fifty-nine patients who underwent laparoscopic resection for right colon cancer were randomized to receive an intracorporeal or an extracorporeal anastomosis (EA). Data including demographics (age, sex, BMI and ASA score), pathological (AJCC tumour stage and tumour localization) and surgical results were recorded. Moreover, to determine the levels of the inflammatory response, mediators, such as C-reactive protein (CRP), tumour necrosis factor (TNF), interleukin 1β (IL-1β), IL-6, IL-10, and IL-13, were evaluated. Similarly, cortisol and insulin levels were evaluated as hormonal responses to surgical stress. We found that the proinflammatory mediator IL-6, CRP, TNF and IL-1β levels, were significantly reduced in IA compared to EA. Concurrently, an improved profile of the anti-inflammatory cytokines IL-10 and IL-13 was observed in the IA group. Relative to the hormone response to surgical stress, cortisol was increased in patients who underwent EA, while insulin was reduced in the EA group. Based on these results, **s**urgical stress and metabolic response to IA justify advocating the adoption of a totally laparoscopic approach when performing a right colectomy for cancer.

This trial is registered on ClinicalTrials.gov (ID: NCT03422588).

## Introduction

Laparoscopic surgery for right-sided colon cancer has achieved a high degree of diffusion in surgical practice and has been widely accepted as an improvement over open procedures^1^. Less is known about the need for fashioning an intracorporeal anastomosis (IA). Even if recent studies and meta-analyses have been published to demonstrate the safety of intracorporeal anastomosis^2^, less is known about the advantages of a totally laparoscopic approach with respect to tissue damage. One of the major advantages could be a better stress response to surgery, but only one recent paper has been published on this topic: in a randomized trial on 60 patients, Mari et al. demonstrated that an IA in laparoscopic right hemicolectomy reduces surgical stress, which may play a role in bowel recovery^3^. This is probably due to the reduced tissue injury and mobilization that characterize the extracorporeal approach. New data introduced in 2018 by Mari and colleagues finds support in the RCTs of Allaix^4^ and Bollo^5^, which have demonstrated superiority of the intracorporeal approach in terms of recovery outcomes; however, surgical stress as a possible reason was not considered. The fundamental role of inflammatory mediators in colorectal surgery had already been hypothesized by Siekmann, whose study aimed to investigate the difference between open and laparoscopic surgery on postsurgical analgesia^6^.

To obtain additional evidence on the choice between these two approaches, we designed this randomized trial in which we evaluated the surgical stress response and the metabolic response in patients who underwent right colonic resection for colon cancer.

## Materials and methods

### Study design

This study was a single-institution, interventional randomized controlled trial designed to compare surgical and stress-related outcomes of patients who underwent laparoscopic right colectomy for colon cancer with intracorporeal (IA) vs extracorporeal anastomosis (EA). This trial is registered on ClinicalTrials.gov (ID: NCT03422588—registration date: 05/02/2018).

### Participants

All consecutive patients ≥ 18 years of age with a histologically confirmed right colonic malignancy planned for elective, segmental, minimally invasive colectomy were recruited. This study adhered to the Declaration of Helsinki and was reviewed and approved by the Ethics Committee of University “Federico II” of Naples. Informed consent was obtained from all enrolled individuals. Patients who planned for open surgery or severe systemic disease that contraindicated minimally invasive surgery and patients who denied consent to participate were excluded from the study.

### Sample size and randomization

The primary end point of the study was postoperative levels of IL-6. To design a study with a 50% or greater minimal predefined reduction in IL-6 levels (30–15 pg/ml) with intracorporeal anastomosis compared to extracorporeal anastomosis, at least 26 participants in each study arm were needed to achieve greater than 80% power with a 5% α error.

Patients were randomized to intracorporeal or extracorporeal groups using computer-based randomization with sealed envelopes. Once the inclusion criteria were met, patients were equally randomized (1:1 allocation) to 1 of 2 groups.

### Outcomes

The data obtained by a competent observer as primary outcome measures, independent of the surgical team, included postoperative interleukin l (IL-6) levels; moreover, as inflammatory mediators, C-reactive protein (CRP), IL-1β, IL-10, IL-13, tumour necrosis factor α (TNFα), cortisol and insulin levels were also recorded.

To determine levels of these markers, whole blood, serum and plasma samples were collected from all recruited subjects. For each participant, fasting samples were collected at the preoperative phase (time 0, T_0_), at 24 h (time 24, T_24_), and 72 h after surgery (time 72, T_72_). The collection tubes were centrifuged at 3000*g* for 10 min, and aliquots of plasma and serum were stored at − 20 °C for subsequent evaluations.

Demographic characteristics [age, sex, body mass index (BMI), American Society of Anesthesiologists (ASA) score and previous abdominal surgery] and pathological features [American Joint Committee on Cancer (AJCC) tumour stage and tumour localization] of all patients were recorded.

Data concerning postoperative surgical complications, such as surgical wound infections, anastomotic leakage, prolonged ileus and abdominal or bowel bleeding, were recorded according to the Clavien-Dindo classification^7^. Anastomotic leakage was defined as a condition of clinical or radiological anastomotic dehiscence that either needed or did not need surgical revision. We considered bleeding for cases that required blood transfusion. Complications were recorded until 30 days after patients’ discharge for all participants.

Regarding recovery, operative time, tolerance to liquid and solid diet, time of first flatus and first stool, mobilization and hospital stay were recorded. Patients were also asked to report their pain on a 10-cm visual analogue scale (VAS), which ranged from 0 for no pain to 10 for severe pain, for their pain 24 and 72 h after surgery.

### Surgical technique

Right colectomy was standardized and performed as previously described^8^.

In detail, the ileocolic pedicles, the right colic vessels and the right branch of the middle colic artery were divided close to their origin intracorporeally. For the EA group, a supraumbilical vertical mini-laparotomy of 6 cm was performed to extract the mobilized bowel using a wound protector. After transection of the colon and ileum, an extracorporeal side-to-side isoperistaltic anastomosis was performed using a mechanical stapler (GIA 100 mm, Medtronic, Minneapolis, MN, USA). The entero-colotomy was closed manually by an interrupted double-layer suture with Vycril 3-0 (Ethicon, Ethicon Inc., Somerville, NJ, USA).

In the IA group, a side-to-side isoperistaltic anastomosis was performed using a mechanical stapler (Echelon Flex 60 mm, Ethicon Endosurgery LLC, Guaynabo, Puerto Rico, USA). The entero-colotomy was closed intracorporeally using a handsewn running barbed double-layer suture with V-Loc 3-0 (Medtronic, Minneapolis, MN, USA). After completion of the anastomosis, the specimen was extracted by a mini-Pfannenstiel incision of 6 cm using a wound protector.

The postoperative period was homogenized, excluding patients receiving different medical and nursing care according to the ERAS protocol^9^. The nasogastric tube was removed at the end of surgery, free fluid oral intake was allowed 6 h after the procedure, and a semisolid diet started on the same day. Early mobilization, starting 4 h postoperatively, was strongly encouraged. Intravenous fluid therapy was stopped the day after surgery, and the urinary catheter was removed. Criteria for discharge included the absence of symptoms, tolerance to a minimum of three meals without restrictions, and passage of stool.

### Determination of inflammatory factors and hormones

Serum levels of CRP were determined following the manufacturer’s protocol using ABX Pentra 400 (Horiba Ltd., Kyoto, Japan). Serum samples were also used for the dosage of the following secreted inflammatory mediators according to the manufacturer’s protocol by Bioplex multiplex Human Cytokine, Chemokine and Growth factor kit (Bio-Rad, Hercules, CA, USA): IL-1β, IL-6, IL-10, IL-13, and TNFα. Serum samples were also assayed for cortisol and insulin levels by automated chemiluminescent immunoassay on the Immulite 2000 Immunoassay system (Siemens AG, Healthcare Sector, Erlangen, Germany).

### Statistical procedures

Statistical analysis was performed using SPSS 23 (SPSS Inc., Chicago, IL, USA). Continuous data are expressed as means ± standard deviation (SD), and categorical variables are expressed as % changes. The Kruskal–Wallis test with a Bonferroni correction was used to analyse categorical data, and the Mann–Whitney test was used to analyse continuous variables.

Baseline characteristics and postoperative variables were compared using multivariate analysis. All results are presented as two-tailed values with statistical significance defined as p-values < 0.05.

## Results

Seventy-two patients who underwent laparoscopic resection for right colon cancer were included in this study. Thirteen patients were excluded from the study for the following reasons: five patients declined to participate in the study, and eight patients needed emergency open surgery. Therefore, a total of 59 patients were eligible and were subsequently randomized: 30 patients were included in the IA group and 29 in the EA group (ESM Appendix 1).

### Population

There were 26/59 males (44%), with a median age of 65.59 ± 3.61 years and a mean BMI of 26.16 ± 3.85 kg/m2. Regarding tumour stage, three patients had AJCC stage 1, 28 patients had AJCC stage 2, 22 patients had AJCC stage 3, and six patients had AJCC stage 4. Regarding localization, in 23 cases, we found caecum cancer; in 21 cases, the tumour was located in the ascending colon; in eight cases tumours were in the hepatic flexure; and in seven cases tumours were in the proximal transverse colon. Comparing IA and EA, we found that the two groups were similar in terms of patient sex, age, BMI, ASA score, AJCC stage and tumour localization (Table 1). All procedures were completed laparoscopically, and no conversions to open surgery were needed.

**Table 1:** Demographic characteristic and recovery outcomes of all randomized patients.

Characteristics	All patients (n = 59)	Intracorporeal anastomosis (n = 30)	Extracorporeal anastomosis (n = 29)	p-value
Male (n, %)	26/59 (44%)	13/30 (43.3%)	13/29 (44.8%)	0.99
Age (years)	65.45 (4.46)	65.26 (4.42)	66.30 (4.19)	0.26
BMI (kg/m^2^)	26.82 (5.67)	26.50 (4.53)	27.31 (6.62)	0.33
**ASA score (n)**	2.91 (0.76)	2.85(0.57)	3.03(0.89)	0.52
II	15	8	7	
III	35	19	16	
IV	9	3	6	
**AJCC Stage (n)**	–	–	–	0.66
I	3	2	1	–
II	28	12	16	–
III	22	13	9	–
IV	6	3	3	–
**Tumor localization (n)**	–	–	–	0.91
Cecum	23	12	11	–
Ascending colon	21	10	11	–
Hepatic flexure	8	4	4	–
Proximal transverse	7	4	3	–
Operative time (min)	134.55 (11.75)	132.35 (9.78)	138.18 (16.12)	0.03
Mobilization (h)	14.11 (3.49)	14.47 (4.36)	13.93 (3.15)	0.73
Time of first flatus (h)	35.44 (14.7)	32.60 (5.18)	46.87 (9.47)	0.001
Time to first stool (h)	65.66 (16.93)	56.26 (6.18)	73.28 (5.51)	0.001
Length of hospital stay (days)	4.92 (0.70)	4.53 (1.01)	5.14 (0.34)	0.001
Tolerance to liquid diet (days)	0.86 (0.13)	0.81 (0.07)	0.92 (0.10)	0.001
Tolerance to solid diet (days)	1.50 (0.23)	1.43 (0.17)	1.62 (0.22)	0.001
VAS Score at 24 h after surgery (h)	5.09 (2.10)	4.19 (2.09)	5.73 (1.23)	0.001
VAS Score at 72 h after surgery (h)	4.80 (1.35)	4.38 (0.8)	5.36 (0.64)	0.001

Data were expressed as median (interquartile range) where nor differently indicated.

*BMI* body mass index, *ASA* American Society of Anesthesiologists, *AJCC* American Joint Committee on Cancer.

### Inflammatory response

**Figure 1:**
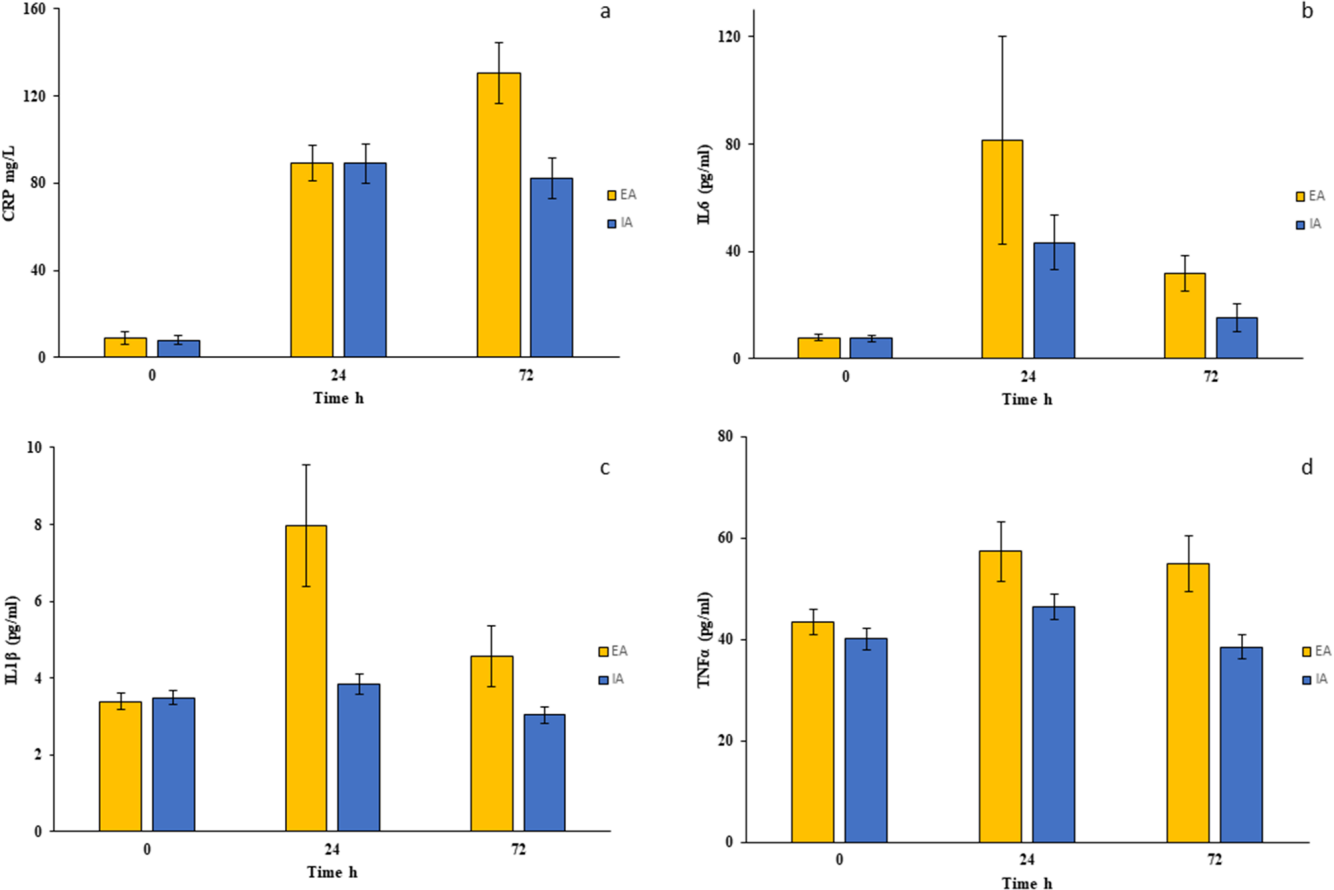
Proinflammatory response after EA and IA surgeries in colon cancer patients. Serum CRP (**a**), IL-6 (**b**), IL-1β (**c**) and TNFα (**d**) levels were evaluated in colon cancer patients undergoing EA or IA anastomosis in laparoscopic right hemicolectomy surgeries 0, 24 and 72 h postsurgery. Results are shown as means ± SD. Statistical analysis was performed using Kurskal Wallis test and Bonferroni post-test correction.

The systemic inflammatory response, in terms of altered secretion of pro- (CRP, IL-6, IL-1β, TNFα) and anti-inflammatory (IL-10 and IL-13) mediators, was investigated pre- (T_0_) and postoperatively (T_24_ and T_72_). At T_0_, no differences in preoperative levels of proinflammatory mediators were observed between the two groups (Fig. 1a–d). In contrast, increased concentrations of CRP were found postoperatively at T_24_ and T_72_ in both the EA and IA groups compared to their respective CRP values at baseline and, comparing the two groups at both T_24_ and T_72_, a significant difference in favour of IA was observed. Similarly, serum levels of IL-6 were increased at T_24_ and T_72_ in both EA and IA groups compared to their respective IL-6 values at baseline, with a significant postoperative reduction in IA compared to EA. Additionally, serum levels of IL-1β were increased postoperatively at T_24_ in the EA group compared to levels at T_0_, while serum concentrations of IL-1β remained unchanged during the entire time course in the IA group. Finally, for TNFα serum levels, there were no differences between groups preoperatively, but at both T_24_ and T_72_, there was a significantly increased concentration in the EA group.

**Figure 2:**
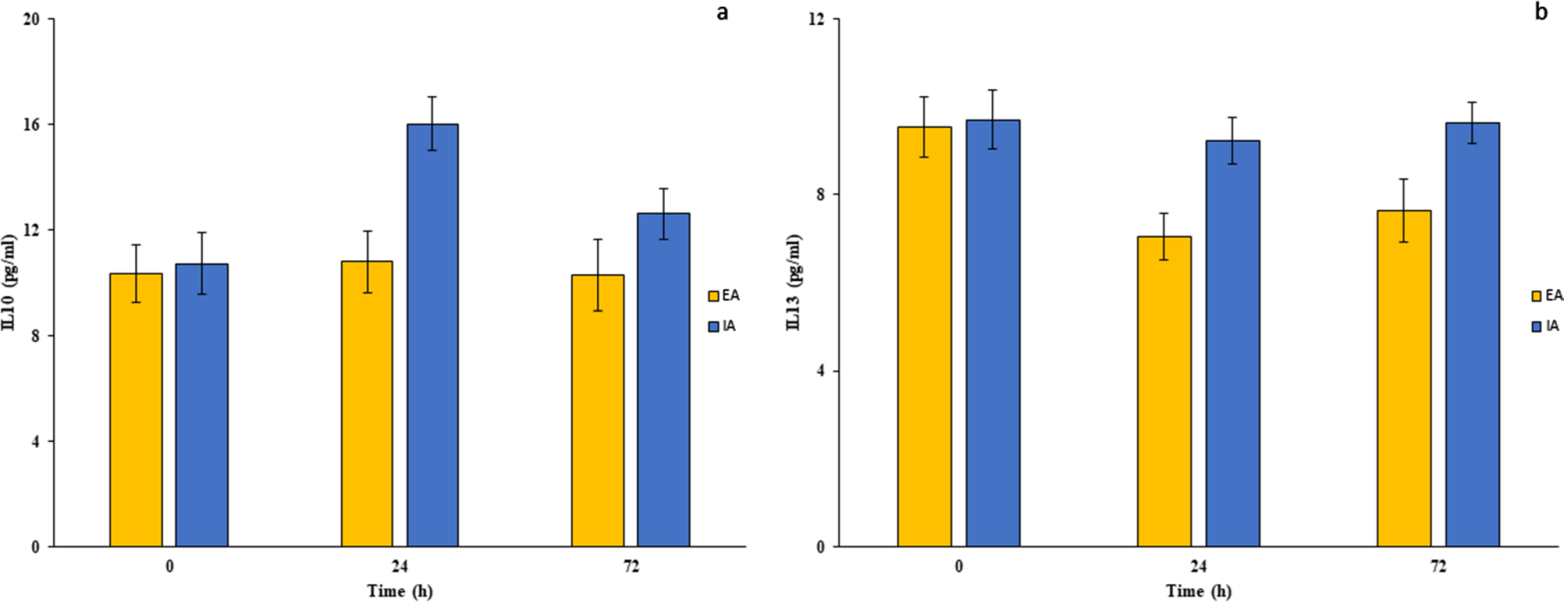
Anti-inflammatory response after EA and IA surgeries in colon cancer patients. Serum IL-10 (**a**) and IL-13 (**b**) levels were evaluated in colon cancer patients undergoing EA or IA anastomosis in laparoscopic right hemicolectomy surgeries 0, 24 and 72 h postsurgery. Results are shown as means ± SD. Statistical analysis was performed using Kurskal Wallis test and Bonferroni post-test correction.

Relative to the anti-inflammatory response, at T_0_, no differences in levels of IL-10 or IL-13 were observed between the two groups (Fig. 2a,b). Serum levels of IL-10 were unchanged postoperatively at T_24_ or T_72_ in the EA group compared to values at baseline, while IL-10 levels were increased at T_24_ and moved towards the baseline concentrations at T_72_ in the IA group. Finally, serum levels of IL-13 were lower at T_24_ and T_72_ in the EA group than at baseline, with a significant reduction compared with IA patients. In contrast, IL-13 levels remained unchanged at T_0_, T_24_ and T_72_ in the IA group (Table 2).

**Table 2:** Inflammatory mediators in all randomized patients.

Characteristics	All patients (n = 59)	Intracorporeal anastomosis (n = 30)	Extracorporeal anastomosis (n = 29)	p-value
**Pre-operative (T**_**0**_**)**				
CRP	8.57 (3.37)	7.85 (3.36)	8.88 (2.51)	0.25
IL-6	7.73 (1.37)	7.53 (1.21)	8.16 (1.48)	0.32
IL-1β	3.36 (0.52)	3.3 (1.78)	3.36 (0.34)	0.59
TNF	42.09 (12.45)	38.78 (14.67)	44.85 (9.97)	0.17
IL-10	10.28 (1.8)	10.6 (2.16)	10.24 (1.12)	0.21
IL-13	9.61 (1.16)	9.53 (1.04)	9.71 (1.28)	0.57
Cortisol	142.6 (17.37)	139.78 (22.71)	143.77 (13.94)	0.44
Insulin	9.04 (1.77)	9.04 (1.85)	9.004 (1.57)	0.89
**24 h after surgery (T**_**24**_**)**				
CRP	88.39 (8.99)	88.84 (8.07)	87.57 (9.94)	0.69
IL-6	55.04 (33.46)	44.24 (8.79)	78.18 (17.7)	0.001
IL-1β	5.55 (4.2)	3.923 (2.22)	8.52 (3.16)	0.001
TNF	45.53 (31.37)	44.28 (27.39)	51.57 (48.9)	0.24
IL-10	13.28 (5.26)	16.2 (1.71)	10.87 (1.44)	0.001
IL-13	8.52 (2.17)	9.17 (0.37)	6.98 (0.74)	0.001
Cortisol	186.03 (44.92)	170.56 (22.01)	215.67 (16.64)	0.001
Insulin	6.2 (1.06)	6.28 (1.1)	6.16 (0.88)	0.87
**72 h after surgery (T**_**72**_**)**				
CRP	99.69 (48.98)	81.94 (10.45)	131.09 (11.78)	0.001
IL-6	17.58 (16.13)	15.32 (1.74)	32.13 (7.46)	0.001
IL-1β	3.9 (1.67)	3.09 (1.54)	4.67 (1.44)	0.001
TNF	47.19 (30.09)	37.1 (18.53)	61.45 (30.23)	0.001
IL-10	11.62 (2.15)	12.62 (1.43)	10.47 (2.21)	0.001
IL-13	8.61 (2.07)	9.63 (0.71)	7.54 (0.64)	0.001
Cortisol	180.69 (30.79)	170.76 (16.03)	199.64 (21.19)	0.001
Insulin	7.18 (3.06)	9.28 (1.27)	6.19 (1.04)	0.001

Data were expressed as median (interquartile range).

*CRP* C-reactive protein, *TNF* tumor necrosis factor, *IL* Interleukin.

### Metabolic stress response

**Figure 3:**
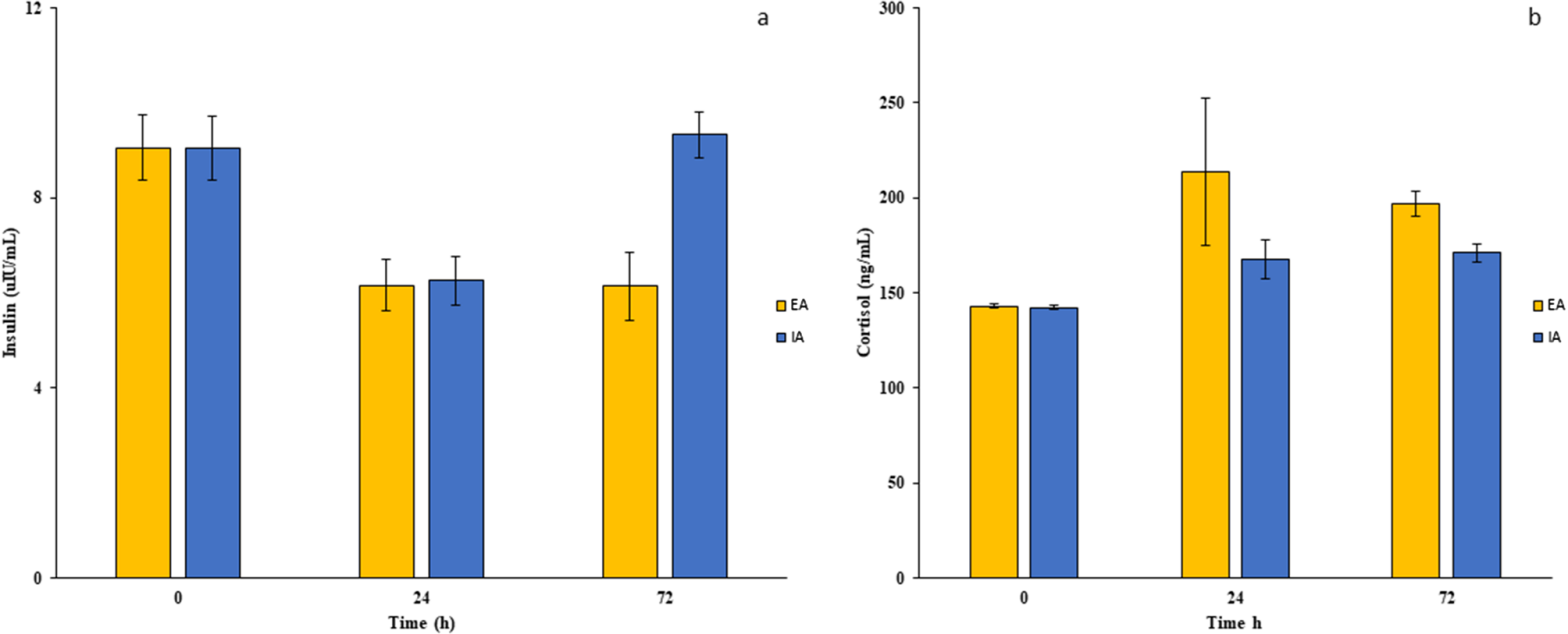
Hormone response after EA and IA surgeries in colon cancer patients. Serum insulin (**a**) and cortisol (**b**) levels were evaluated in colon cancer patients undergoing EA or IA anastomosis in laparoscopic right hemicolectomy surgeries at time 0 h and at 24 and 72 h postsurgery. Results are shown as means ± SD. Statistical analysis was performed using Kurskal Wallis test and Bonferroni post-test correction.

The metabolic stress response was also evaluated in terms of altered secretion of hormones, such as cortisol and insulin, in colon cancer patients undergoing EA or IA surgeries. At T_0_, no differences in preoperative levels of insulin and cortisol were observed between the two groups (Fig. 3a,b). Cortisol levels were increased postoperatively at T_24_ and T_72_ in the EA groups compared to baseline values. On the other hand, no significant increase in this hormone was observed postoperatively compared to T_0_ in the IA group (Fig. 3a). Insulin levels were reduced postoperatively at T_24_ and T_72_ in the EA group and only at T_24_ in the IA group compared to respective insulin values at T_0_ (Table 2).

### Complications

Between randomized subjects, 11 patients had postoperative complications. We recorded 1 anastomotic leak, 2 anastomotic bleeds and 1 ileus in the IA group. In the EA group, we recorded one anastomotic leak, one anastomotic bleed, one wound infection and four ileus. Statistical analysis revealed no differences between the two groups (IA: Clavien-Dindo 1: one case—Clavien-Dindo 2: two cases—Clavien-Dindo 3: two cases; EA: Clavien-Dindo 1: three case—Clavien-Dindo 2: three cases—Clavien-Dindo 3: one cases; p = 0.53). All complications occurred between postoperative day (POD) 1 and 30 days after discharge.

### Recovery

Differences were found in recovery outcomes in favour of patients who underwent IA with respect to operative time, time of first flatus, time of first stool, length of hospital stay, tolerance to liquid diet and solid diet. Furthermore, VAS score 24 and 72 h after surgery was significantly lower in the IA group compared to the EA group. These results are shown in Table 1.

### Additional analysis in uncomplicated patients

Among patients without complications, there were 21/48 males (43.7%), with a median age of 65.80 ± 3.8 years and a mean BMI of 26.24 ± 3.9 kg/m^2^. Regarding tumour stage, three patients had AJCC stage 1, 21 patients had AJCC stage 2, 19 patients had AJCC stage 3, and five patients had AJCC stage 4. Moreover, six patients had undergone previous abdominal surgery. Regarding localization, in 18 cases, we identified caecum cancer; in 19 cases, the tumour was located in the ascending colon, in six cases in the hepatic flexure and in five cases in the proximal transverse colon. Comparing IA and EA, we found that the two groups were not significantly different in terms of sex, age, BMI, ASA score, AJCC stage and tumour localization (Table 3).

**Table 3:** Demographic characteristic and recovery outcomes of patients without post-operative complications.

Characteristics	All patients (n = 48)	Intracorporeal anastomosis (n = 24)	Extracorporeal anastomosis (n = 24)	p-value
Male (n, %)	21/48 (43.7%)	13/24 (54.1%)	8/24 (33.3%)	0.24
Age (years)	66.26 (4.71)	65.31 (5.43)	66.65 (4.10)	0.37
BMI (kg/m^2^)	26.73 (5.85)	25.78 (4.65)	27.44 (6.92)	0.13
**ASA score (n)**	2.94 (0.72)	2.87 (0.58)	3 (0.8)	0.78
II	12	5	7	
III	29	16	13	
IV	7	3	4	
**AJCC Stage (n)**	–	–	–	0.38
I	3	2	1	–
II	21	12	16	–
III	19	13	9	–
IV	5	3	3	–
**Tumor localization (n)**	–	–	–	0.96
Cecum	18	12	11	–
Ascending colon	19	10	11	–
Hepatic flexure	6	4	4	–
Proximal transverse	5	4	3	–
**Operative time (min)**	133.87 (11.84)	132.12 (9.18)	138.05 (16.25)	0.03
Mobilization (h)	13.68 (4.03)	13.85 (5.27)	13.68 (3.73)	0.73
Time of first flatus (h)	37.3 (14.31)	32.96 (5.13)	47.21 (9.71)	0.001
Time to first stool (h)	65.82 (17.11)	55.89 (5.66)	73.19 (6.53)	0.001
Length of hospital stay (days)	4.89 (0.82)	4.27 (0.71)	5.09 (0.29)	0.001
Tolerance to liquid diet (days)	0.86 (0.13)	0.82 (0.08)	0.92 (0.13)	0.001
Tolerance to solid diet (days)	1.5 (0.21)	1.44 (0.17)	1.61 (0.20)	0.001
VAS Score at 24 h after surgery (h)	5.1 (1.8)	4.19 (1.89)	5.66 (1.31)	0.001
VAS Score at 72 h after surgery (h)	4.85 (1.32)	4.35 (0.74)	5.36 (0.76)	0.001

Data were expressed as median (interquartile range) where not differently indicated.

*BMI* body mass index, *ASA* American Society of Anesthesiologists, *AJCC* American Joint Committee on Cancer.

In terms of the inflammatory response, we similarly found that serum levels of CRP and IL-6 were increased at T_24_ and T_72_ in both the EA and IA groups compared to their respective values at baseline, with a significant postoperative reduction in IA at T_72_. Levels of IL-1β were increased at T_24_ in the EA group, while in IA, its concentrations remained unchanged, and for TNFα serum levels at both T_24_ and T_72_, there was an increased concentration that was significantly higher in the EA group. Serum levels of IL-10 were unchanged postoperatively at T_24_ and T_72_ in the EA group but were increased at T24 and had moved towards the baseline concentrations at T_72_ in the IA group. IL-13 was lowered at T_24_ and T_72_ in the EA group, while its levels remained unchanged at T_0_, T_24_ and T_72_ in the IA group. Regarding the metabolic stress response, cortisol levels were postoperatively increased at T_24_ and T_72_ in the EA group but were not increased in the IA group. Finally, insulin levels were reduced at T_24_ and T_72_ in the EA group and only at T_24_ in the IA group (Table 4).

**Table 4:** Inflammatory mediators in patients without post-operative complications.

Characteristics	All patients (n = 48)	Intracorporeal anastomosis (n = 24)	Extracorporeal anastomosis (n = 24)	p-value
**Pre-operative (T**_**0**_**)**				
CRP	8.43 (3.51)	7.75 (3.15)	8.77 (3.42)	0.24
IL-6	7.68 (1.57)	7.34 (0.99)	8.23 (1.54)	0.28
IL-1β	3.3 (0.52)	3.12 (1.32)	3.35 (0.33)	0.19
TNF	41.49 (11.69)	39.07 (14.98)	42.33 (9.63)	0.57
IL-10	10.28 (1.86)	10.45 (2.44)	10.25 (1.2)	0.33
IL-13	9.58 (1.16)	9.53 (1.38)	9.63 (1.1)	0.30
Cortisol	143.03 (17.66)	138.32 (22.58)	145.59 (14.06)	0.39
Insulin	8.95 (1.28)	8.81 (1.54)	9 (1.38)	0.81
**24 h after surgery (T**_**24**_**)**				
CRP	87.62 (8.4)	88.84 (7.51)	86.81 (9.86)	0.27
IL-6	55.71 (32.36)	44.79 (8.65)	77.53 (18.16)	0.001
IL-1β	5.58 (4.58)	3.93 (1.93)	8.54 (2.79)	0.001
TNF	49.9 (33.7)	48 (28.48)	50.15 (55.99)	0.37
IL-10	13.1 (5.38)	16.26 (1.58)	10.81 (1.07)	0.001
IL-13	8.47 (2.27)	9.17 (0.45)	6.89 (0.79)	0.001
Cortisol	190.81 (46.2)	168.77 (20.53)	215.75 (15.53)	0.001
Insulin	6.16 (0.95)	6.19 (0.99)	6.16 (0.87)	0.95
**72 h after surgery (T**_**72**_**)**				
CRP	104.78 (52.23)	79.28 (8.7)	132.09 (12.53)	0.001
IL-6	17.26 (15.86)	15.24 (1.4)	31.46 (6.98)	0.001
IL-1β	3.91 (1.89)	2.78 (2.09)	4.67 (1.53)	0.001
TNF	48.8 (27.86)	40.89 (16.19)	60.61 (26.96)	0.006
IL-10	11.7 (2.12)	12.53 (1.35)	10.41 (2.04)	0.001
IL-13	8.64 (2.02)	9.63 (0.67)	7.59 (0.8)	0.001
Cortisol	180.14 (36.34)	169.22 (13.54)	203.64 (24.55)	0.001
Insulin	7.09 (3)	9.2 (1.56)	6.15 (0.8)	0.001

Data were expressed as median (interquartile range).

*CRP* C-reactive protein, *TNF* Tumor Necrosis Factor, *IL* Interleukin.

Recovery outcomes confirmed significantly better results in favour of IA in terms of operative time, time of first flatus, time of first stool, length of hospital stay, tolerance to liquid diet and solid diet, and VAS score 24 and 72 h after surgery (Table 3).

### Additional analysis in complicated patients

Between patients who experienced complications, there were 5/11 males (45.4%), with a median age of 64.67 ± 2.54 years. Mean BMI was 25.79 ± 3.75 kg/m^2^. Regarding tumour stage, three patients had AJCC stage 2, six patients had AJCC stage 3, and two patients had AJCC stage 4. Moreover, one patient had undergone previous abdominal surgery. Regarding localization, in five cases, we identified caecum cancer; in two cases, the tumour was located in the ascending colon, in two cases in the hepatic flexure and in two cases in the proximal transverse colon. Comparing IA and EA, we found that the two groups were not significantly different in terms of age, BMI, ASA score, AJCC stage and tumour localization (Table 5).

**Table 5:** Demographic characteristic and recovery outcomes of patients with post-operative complications.

Characteristics	All patients (n = 11)	Intracorporeal anastomosis (n = 6)	Extracorporeal anastomosis (n = 5)	p-value
Male (n, %)	5/11 (45.45%)	0	5/5 (100%)	0.001
Age (years)	64.83 (2.58)	64.70 (1.91)	64.83 (2.97)	0.37
BMI (kg/m^2^)	26.91 (4.87)	27.09 (3.92)	25.88 (6.25)	0.32
**ASA score (n)**	–	–	–	0.53
II	3	3	0	
III	6	3	3	
IV	2	0	2	
**AJCC stage (n)**	–	–	–	0.78
II	7	4	3	–
III	3	1	2	–
IV	1	1	0	–
**Tumor localization (n)**	–	–	–	0.95
Cecum	5	3	2	–
Ascending colon	2	1	1	–
Hepatic flexure	2	1	1	–
Proximal transverse	2	1	1	–
Operative time (min)	136.16 (8.65)	133.63 (12.19)	140.45 (10.22)	0.001
Mobilization (h)	14.90 (1.98)	14.97 (1.82)	14.11 (1.58)	0.81
Time of first flatus (h)	33.10 (15.47)	30.42 (3.77)	46.55 (3.54)	0.001
Time to first stool (h)	64.57 (17.48)	56.62 (3.38)	76.11 (7.17)	0.001
Length of hospital stay (days)	5.43 (0.17)	5.53 (0.42)	5.40 (0.06)	0.001
Tolerance to liquid diet (days)	0.88 (0.18)	0.81 (0.09)	1.0 (0.13)	0.001
Tolerance to solid diet (days)	1.56 (0.26)	1.46 (0.22)	1.74 (0.18)	0.001
VAS Score at 24 h after surgery (h)	5.05 (2.54)	4.16 (2.05)	6.16 (0.45)	0.001
VAS Score at 72 h after surgery (h)	4.58 (1.34)	4.48 (1.10)	5.53 (1.32)	0.001

Data were expressed as median (interquartile range) where not differently indicated.

*BMI* body mass index, *ASA* American Society of Anesthesiologists, *AJCC* American Joint Committee on Cancer.

In terms of the inflammatory response, we similarly found that serum levels of CRP and IL-6 were increased at T_24_ and T_72_ in both the EA and IA groups compared to their respective values at baseline, with a significantly higher increase in EA at T_72_. Levels of IL-1β were increased at T_24_ in the EA group, while in IA, its concentrations remained unchanged, and TNFα serum levels at both T_24_ and T_72_ remained stable in both groups. Serum levels of IL-10 were unchanged postoperatively at T_24_ and T_72_ in the EA group but were increased at T_24_ and moved towards the baseline concentrations at T_72_ in the IA group. Regarding the metabolic stress response, cortisol levels were increased postoperatively at T_24_ and T_72_ in both groups, but they were significantly higher in EA patients. Finally, insulin levels were reduced at T_24_ and T_72_ in the EA group and only at T_24_ in the IA group (Table 6).

**Table 6:** Inflammatory mediators in patients with post-operative complications.

Characteristics	All patients (n = 59)	Intracorporeal anastomosis (n = 30)	Extracorporeal anastomosis (n = 29)	p-value
**Pre-operative (T**_**0**_**)**				
CRP	8.90 (1.68)	8.58 (2.51)	9.06 (1.90)	0.08
IL-6	7.81 (0.77)	7.71 (0.75)	8.0 (0.43)	0.28
IL-1β	3.62 (1.21)	4.57 (1.43)	3.51 (0.28)	0.71
TNF	44.30 (13.08)	37.91 (16.14)	45.72 (4.58)	0.25
IL-10	11.36 (2.01)	11.70 (2.14)	10.19 (1.22)	0.29
IL-13	9.82 (1.28)	9.59 (0.57)	10.21 (2.49)	0.46
Cortisol	141.46 (15.16)	143.70 (18.32)	138.52 (7.37)	0.81
Insulin	10.26 (2.13)	10.28 (1.56)	9.46 (2.43)	0.91
**24 h after surgery (T**_**24**_**)**				
CRP	90.04 (5.67)	88.86 (7.79)	93.06 (3.63)	0.89
IL-6	46.25 (43.38)	40.78 (8.82)	91.11 (15.34)	0.001
IL-1β	5.53 (2.34)	4.17 (2.24)	6.69 (3.13)	0.001
TNF	39.76 (12.09)	39.67 (3.58)	51.73 (15.10)	0.19
IL-10	14.48 (3.99)	15.97 (2.17)	11.49 (2.51)	0.001
IL-13	9.02 (1.97)	99.25 (0.26)	7.19 (0.40)	0.001
Cortisol	184.2 (22.99)	177.50 (20.40)	202.05 (18.79)	0.001
Insulin	6.28 (1.05)	6.56 (1.31)	6.16 (1.10)	0.72
**72 h after surgery (T**_**72**_**)**				
CRP	99.69 (41.53)	86.56 (2.21)	130.76 (11.35)	0.001
IL-6	17.86 (18.79)	15.81 (1.47)	38.80 (8.81)	0.001
IL-1β	3.90 (1.04)	3.42 (0.54)	4.66 (0.91)	0.001
TNF	35.56 (25.17)	30.77 (12.08)	63.0 (30.11)	0.001
IL-10	11.53 (2.99)	13.90 (2.30)	11.03 (3.22)	0.001
IL-13	8.53 (2.30)	9.56 (0.64)	7.08 (0.43)	0.001
Cortisol	182.93 (17.37)	177.84 (11.46)	195.91 (11.54)	0.001
Insulin	7.78 (2.61)	9.48 (1.17)	6.81 (1.38)	0.001

Data were expressed as median (interquartile range).

*CRP* C-reactive protein, *TNF* tumor necrosis factor, *IL* interleukin.

Recovery outcomes confirmed significantly better results in favour of IA with respect to operative time, time of first flatus, time of first stool, length of hospital stay, tolerance to liquid diet and solid diet, and VAS score 24 and 72 h after surgery (Table 5).

## Discussion

The first minimally invasive colon resection was described in 1991 by Jacobs et al.^10^, and after 30 years, the present literature indicates that a minimally invasive approach could be considered the gold standard for performing a right colectomy in patients with cancer.

Less consensus has been achieved when choosing whether to perform an intracorporeal or an extracorporeal anastomosis after laparoscopic right colectomy. Many surgeons still prefer to fashion an EA^11^, likely due to the technical difficulties related to IA and the need to perform, in most cases, laparoscopic hand-sewn sutures^12^. Nevertheless, evolution of the surgical technique towards a minimally invasive approach has justified the increasing adoption of IA for totally laparoscopic procedures^13–19^.

Several studies have been published that demonstrate the safety of IA. In a study on 597 patients who underwent laparoscopic right colectomy, Bou Saleh et al.^20^ found that fashioning an IA results a lower complication rate with a shorter hospital stay compared to an open approach. Cleary et al.^8^ analysed results from 1029 subjects who underwent right colectomies, recording that the IA group had a significantly lower conversion to open rate, shorter hospital length of stay and lower complication rate. In an RCT with 60 patients, Vignali et al.^21^ found an earlier recovery of bowel function and a lower incidence of postoperative ileus with IA. Finally, Aiolfi et al.^22^, in a recent meta-analysis including 23 studies and 3755 patients, underlined that IA seems to be associated with reduced postoperative infections, overall complications and recovery parameters.

With this point of view, one of the hypothesized advantages of a total minimally invasive approach could be considered the reduction of stress response to surgery and surgical trauma. Recent literature has demonstrated that a major factor in the development of morbidity is the surgical stress response with subsequent increased demand on the patient’s reserves and immune competence^23,24^.

Surgical stress plays a key role in recovery outcomes. The body’s reaction to surgical trauma begins with activation of the hypothalamic–pituitary–adrenal axis. This leads to an endocrine response with the release of hormones, such as cortisol, growth hormone, and prolactin, in the bloodstream. The effect on the adrenal glands leads to catecholamine synthesis, which is responsible for tachycardia, vasoconstriction, and hyperglycaemia. Proinflammatory cytokines are locally produced by the injured tissue as a direct consequence of trauma. This has both local and systemic consequences^25^.

In a randomized trial that compared the surgical stress response between laparoscopic and open colon cancer surgery, Veenhof et al.^24^ found that there were lower levels of IL-6, CRP and growth hormone in patients who underwent laparoscopic surgery with fast-track postoperative care. Pappas-Gogos et al.^26^ randomized 60 patients with colorectal cancer to laparoscopic and open groups, measuring oxidative stress markers. They found that postoperative serum levels of 8-epiPGF2a, 3-NT, and 8-OHG were significantly lower in the laparoscopic group at all times of detection. Similarly, Shibata et al.^27^ compared 46 colorectal surgery patients who underwent robotic, laparoscopic and open resection and found that perioperative surgical stress in terms of HLA-DR (marker of immune competence), CRP levels and lymphocyte subset counts (natural killers, cytotoxic T cells and helper T cells) was comparable between robotic and laparoscopic surgery and was higher in response to open surgery. Finally, Nishiguchi et al.^28^ evaluated the benefits of laparoscopic procedures for colorectal carcinoma by analysing IL6 and CRP levels after laparoscopic and open surgery and found that both markers were significantly greater in the open group than in the laparoscopic group one day and two days after surgery.

In contrast, less is known about the differences in surgical stress response when an IA or EA is chosen during laparoscopic colon cancer surgery. Previous literature is represented only by the study of Mari et al.^3^, who found that IL-6 and CRP levels were significantly lower in the IA group postoperatively than in the EA group.

Their results suggest that surgical and metabolic response to an IA justify the adoption of an entirely laparoscopic approach performing a right colectomy for cancer, but the role of other inflammatory mediators, such as IL-1β, IL-10, IL-13, TNFα and insulin, was not considered.

Herein, we designed a randomized trial to evaluate the surgical stress response and the metabolic response in patients who underwent right colonic resection for colon cancer; we chose IL-6 as the primary outcome because, as already demonstrated, its production is a direct consequence of tissue trauma, and it is synthetized locally and acts as a trigger for liver synthesis of other acute phase proteins, such as CRP.

Our results demonstrated that all parameters related to surgical stress were lower in the intracorporeal approach to anastomosis. We found that the systemic inflammatory response, in terms of altered secretion of proinflammatory mediators, was reduced in patients who underwent IA compared to those who underwent EA. Indeed, postoperatively, CRP, TNFα, IL-6 and IL-1β levels were all reduced. Concurrently, an improved profile of the anti-inflammatory cytokines IL-10 and IL-13 was observed in the IA group. Relative to the hormone response to surgical stress, cortisol was found to be increased in patients who underwent EA anastomosis, while insulin was found to be reduced in the EA group. Thus, reduced levels of cortisol, CRP, IL-6, and IL-1β accompanied by the recovery of insulin levels and anti-inflammatory mediators may predispose patients to a reduced incidence of postoperative complications.

These results might also reflect a biological underpinning to the longstanding question of why patients undergoing entirely laparoscopic techniques with IA have been described to exhibit accelerated recovery^29,30^. From this point of view, we found IA was associated to a 30% decrease of time of first flatus and first stool and to a 12% decrease of hospital stay; moreover, this approach was associated to a 30% decrease of postoperative pain at 24 and 72 h after surgery.

This lower perception of pain reduced analgesic requirements, and rapid mobilization and regular food intake resulted in a shorter stay for patients who perceived improved postsurgical recovery and were able to resume daily activities.

Considering these results in conjunction with our findings about inflammatory mediators and metabolic changes, it is reasonable to assume that surgical stress has an important impact on recovery outcomes. Therefore, it is reasonable to declare that the reduced surgical stress related to the choice of an IA is correlated with faster recovery after surgery. It is clear, however, that although the statistics are significant, the differences found between the two groups are not so relevant from a clinical point of view; in this perspective, further studies with a larger sample size will be able to better define the clinical impact of these two approaches.

## Supplementary Information

Supplementary Information.
