# Supplementary material for: Surgical stress and metabolic response after totally laparoscopic right colectomy
Source: Sci Rep. 2021 May 6;11:9652. doi: 10.1038/s41598-021-89183-7 (PMC8102592; doi:10.1038/s41598-021-89183-7)
Supplement: Supplementary file 1 — Supplementary Information. [file 41598_2021_89183_MOESM1_ESM.docx]

**Surgical stress and metabolic response after totally laparoscopic right colectomy**

Marco Milone*, MD, PhD^1,¶^, Antonella Desiderio*, PhD^2,3,¶^, Nunzio Velotti^4^,MD, Michele Manigrasso^4^,MD, Sara Vertaldi^1^,MD, Umberto Bracale^1^,MD, Michele D’Ambra^1^,MD, Giuseppe Servillo, MD^1^, Giuseppe De Simone^1^, MD, Fatima Domenica Elisa De Palma^5^,MD, Giuseppe Perruolo^2,3^,MD, Gregory A Raciti, PhD ^2,3^, Claudia Miele, PhD, ^2,3^ Francesco Beguinot, MD, PhD,^2,3^, Giovanni Domenico De Palma^1^, MD.

^1^ Department of Clinical Medicine and Surgery, “Federico II” University of Naples, Naples, Italy;

^2^ URT “Genomics of Diabetes”, Institute of Experimental Endocrinology and Oncology “G. Salvatore”, National Council of Research, Naples, Italy;

^3^ Department of Translational Medical Sciences, “Federico II” University of Naples, Naples, Italy;

^4^ Department of Advanced Biomedical Science, “Federico II” University of Naples, Naples, Italy;

^5^ CEINGE-Biotecnologie avanzate. INSERM U1138, Centre de Recherche des Cordeliers, Sorbonne Université, Université de Paris, Paris;France; Team “Metabolism, Cancer & Immunity”, Equipe 11.

**CONSORT 2010 Flow Diagram**

Excluded (n=13)

- Declined to participate (n=5)
- Needed emergency open surgery (n=8)

Analysed (n=30)

Analysed (n=29)

## Allocation

## Analysis

Allocated to extracorporeal anastomosis (n=29)

♦ Received allocated intervention (n=29)

Allocated to intracorporeal anastomosis (n=30)

♦ Received allocated intervention (n=30)

## Enrollment

Randomized (n=59)

Assessed for eligibility (n=72)
